# Reaching Into the Unknown: Actions, Goal Hierarchies, and Explorative Agency

**DOI:** 10.3389/fpsyg.2018.00266

**Published:** 2018-03-07

**Authors:** Davood G. Gozli, Nevia Dolcini

**Affiliations:** ^1^Department of Psychology, Faculty of Social Sciences, University of Macau, Macau, China; ^2^Philosophy and Religious Studies Programme, Faculty of Arts and Humanities, University of Macau, Macau, China

**Keywords:** agency, control, exploration, exploitation, goal, goal-directed action, improvisation

## Abstract

Action is widely characterized as possessing a teleological dimension. The dominant way of describing goal-directed action and agency is in terms of *exploitation*, i.e., pursuing pre-specified goals using existing strategies. Recent theoretical developments emphasize the place of *exploration*, i.e., discovering new goals or acquiring new strategies. The exploitation-exploration distinction poses questions with regard to goals and agency: Should exploration, as some authors have suggested, be regarded as acting without a goal? We argue that recognizing the hierarchical nature of goals is crucial in distinguishing the two kinds of activity, because this recognition prevents the claim that exploration is goal-free, while allowing for a homogeneous account of both exploitative and explorative actions. An action typically causes relatively low-level/proximal (i.e., sensorimotor, immediate) and relatively high-level/distal (i.e., in the environment, at a wider timescale) outcomes. In exploitation, one relies on existing associations between low- and high-level states, whereas in exploration one does not have the ability or intention to control high-level/distal states. We argue that explorative action entails the capacity to exercise control within the low-level/proximal states, which enables the pursuit of indeterminate goals at the higher levels of a goal hierarchy, and the possibility of acquiring new goals and reorganization of goal hierarchies. We consider how the dominant models of agency might accommodate this capacity for explorative action.

The present article is concerned with the distinction between two kinds of activities, so-called *exploitation* and *exploration* (Hills et al., 2015). Exploitation refers to activities directed at specific goals and relying on existing strategies. Exploration refers to activities that are either not directed at a specific goal or deviate from existing strategies^1^. Our argument is that framing the distinction in terms of presence *vs*. absence of goals is not satisfactory: deprived of goals, exploration would hardly qualify as genuine action. Our argument is grounded in the following three premises. First, goals are hierarchically organized. Second, exploration implies goals at the lower levels of a goal hierarchy, although those lower-level goals are not tightly attached to particular higher-level goals.Third, exploration is a generative capacity, for the acquisition of new goals and for the reorganization of goal hierarchies.

## Hierarchy of goals: low-level (L-) and high-level (H-) goals

The hierarchical organization of goals becomes apparent when we point out that the same action can be described in terms of different outcomes (e.g., Anscombe, 1957; Chambon et al., 2017). An action can be described as “manually flipping a switch,” “turning on a light bulb,” “making the room brighter,” and “making one's reading experience more comfortable.” While the first two descriptions refer to the performance and the tools of action, the latter refer to the room, the visibility of objects, and a wider context of activity. This set of descriptions range, respectively, from relatively proximal to relatively distal outcomes (Mele, 2000; Pacherie, 2008). Within a hierarchy of goals, they range from goals at the relatively lower levels of the goal hierarchy (hereafter, L-goals), e.g., movements in the body, to goals at the relatively higher levels (H-goals), e.g., maintaining the brightness level of the room such that one can continue reading (Powers, 1998; Chambon et al., 2011a).

An agent, for whom goals can be identified at multiple levels, can intend outcomes at multiple levels. Consider going home from work. At one level, this involves fulfilling the goal of arriving home (H-goal). At another level, it involves enacting a sequence of navigations (L-goals). Consider, furthermore, telling a joke to a group of friends. At one level, this involves fulfilling the goal of making your friends laugh (H-goal). At another level, it involves enacting a sequence of utterances and gestures. The skills involved at the level of L-goals could be used to fulfill different H-goals. You could employ the same skills that enabled one sequence of navigations to arrive at a different destination; and, you could employ the same skills that enabled telling the joke to share a bad news. Nonetheless, in a specific instance, L-goals and H-goals are integral parts of the same action, which is to say that the action would change if either of the goals change. You might go home by driving, walking, or taking the helicopter. Although these actions all equally fulfill the higher-level intention (i.e., to go home), they are not the same actions. The teleological dimension of an action is, therefore, intended as a set of goals that are hierarchically organized.

Related to the notion of goal hierarchies are two additional concepts of agency and control. We use *agency* to refer to the agent's awareness that an outcome resulted from her own action (Wolpert, 1997), while we use *control* to refer to the agent's awareness of the contingencies that link movements and outcomes. A person pushes a button without knowing the consequence, and observers a light turning on. Here, the action involves agency (“I turned on the light!”) but not control (“I didn't know the button was linked to the light!”). Regular co-occurrence of proximal outcomes (L-goals) with relatively distal outcomes can result in forming new associations and, consequently, acquiring the ability to intentionally use the distal outcomes as H-goals (Elsner and Hommel, 2001).

Applying the concept of control to goal hierarchies reveals the possibility of having control over a specific action at one level (L-goals), yet not having control at another level (H-goal). It is possible to have full control over a sequence of navigation while lacking control over the final destination; it is possible to have full control over one's utterances and gestures without knowing the listeners' response. These distinctions are crucial in understanding the difference between exploitative and explorative action, as well as in revealing essential (underplayed) features of exploration.

## Regarding goal as a unitary concept

The idea that goals are hierarchically organized is not controversial, but empirical research on action tends to neglect the hierarchical nature of goals (for exceptions see, e.g., Chambon et al., 2011a,b, 2017). The goal of an action is typically regarded as unitary, and in terms of one level in the hierarchy.

A study by Borhani et al. (2017) provides an illustration of this point. The researchers were interested in examining two factors in determining sense of agency. Using a button-press task, they examined the role of having choice over which button is pressed (factor 1) and whether or not one performs the chosen button-press oneself (factor 2). That is, in some conditions participants selected the to-be-pressed button and in other conditions they were instructed to press the button. Furthermore, in some conditions participants pressed the chosen button themselves and in other conditions an experimenter pressed their fingers against the button. At first glance, it may appear that factors 1 (choice of outcome) and 2 (motoric control) are manipulated independently of each other. And, indeed, the authors claimed that the two factors are independent. In addition, only factor 1 was regarded as having to do with the outcome (i.e., that which can serve as a goal) of the actions.

From the present perspective, the two factors correspond to H- and L-goals. It is not the case that the action-outcome in this task is only identifiable as the choice over the button or the final state in which a button is pushed. The sensorimotor state that accompanies the button-press is also an identifiable outcome. While the tactile pressure against a specific fingertip as a result of voluntary movement is an action-outcome, a similar tactile sensation that results from forced movement is not an action-outcome. Thus, the same higher-level outcome (e.g., a button being pushed) can be regarded as a goal in only one of the two conditions. Given that both H- and L-goals are integral to each specific action, the two factors cannot be manipulated independently. This is particularly salient in Borhani et al.'s (2017) study, as the consequence of a button-press was the delivery of a painful stimulus, the level of which depended on the button.

Compare, for instance, performing a voluntarily chosen key-press that delivers a relatively high-intensity painful stimulus with performing the same key-press following instruction. The two acts differ not only in their meaning within the context of the experimenter-participant relationship, but also very likely in terms of their sensorimotor characteristics (Janczyk et al., 2015; Hommel et al., 2017). It is, therefore, unsurprising that the two factors in Borhani et al.'s (2017) did not have independent effects. They found the strongest evidence for the sense of agency in the condition where the button was voluntarily chosen and the button-press was enacted, such that the combined effect of the two factors was more than the sum of their individual effects.

Another series of studies by Wen et al. (2015) provides an illustration of how researchers tend to regard goals as unitary and non-hierarchical. The researchers were interested in understanding the role of goals in the sense of agency. They used a movement task, in which participants had varying degrees of control over a moving dot on the computer screen. In some conditions, a small static square on the screen served as goal location and the participants tried to hit the square with the dot as many times as possible (“goal condition”). In the other conditions, the small square was absent and the participants simply tried to move the dot in any direction they wished (“no-goal condition”). Wen et al. (2015, 2016) found stronger sense of agency in the “no-goal condition” relative to the “goal condition.”

From the present perspective, the “goal condition,” in Wen et al.'s studies, involves both an H-goal (hitting the target) and L-goals (controlling the moving dot). In comparison, the “no-goal condition” may not have an H-goal, aside from the rather vague goal of affecting the moving dot, but it does involve L-goals. When describing the task, the L-goals are taken for granted and their status as L-goals, within a hierarchy of goals, is neglected (Gozli, 2017). This bias might result from our common intuition that actions aim at easily and publicly identifiable goals (e.g., hitting the target). Such goals are identified at a particular level of the goal hierarchy that is neither too high-level (e.g., “be a good person”) nor too low-level (e.g., “make an eye-movement”), with the result that other integral goals at other levels are neglected, yet present. Regarding goals as unitary is reflected in theories of goal-directed action, to which we now turn.

## Understanding explorative activity

Neglecting the hierarchical nature of goals and, subsequently, regarding an action's goal in terms of a single level, reinforces a dichotomy between (goal-directed) exploitation and exploration. Hills et al., 2015 wrote that “taking too much time to deliberate can be disastrous. At some point, the search (exploration) has to be stopped and the action (exploitation) taken.” (p. 50) This implies that the dichotomy is partly in terms of the presence/absence of goals.

**Figure 1:**
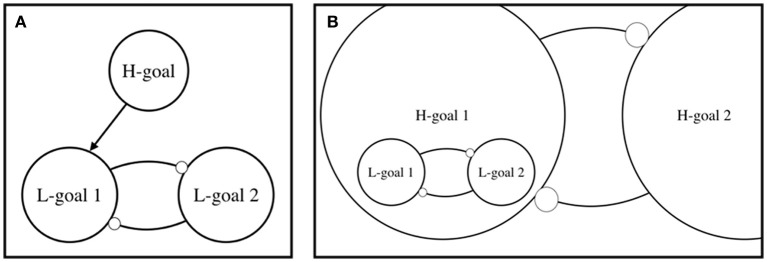
A simple model of goal-directed activity in which lower-level (L-)goals are actualized in accordance with higher-level (H-)goals (cf., Hommel, 2015; Hommel and Colzato, 2017). The panel **(A)** closely follows Hommel (2015), and makes a clear distinction between the different levels. The panel **(B)** emphasizes that the same logic applies to goals at different levels of the hierarchy, and that H-goals are constituted in part by their implementation in terms of L-goals, rather than being an external factor to them. For the sake of simplicity, we have included only two levels in the hierarchy.

A model of action control, which provides an account of explorative activity, has been put forth by Bernhard Hommel (e.g., Hommel, 2015; Hommel and Colzato, 2017; see also, Goschke, 2013). The model's essential parameters are shown in Figure 1A. This simple version aims to capture action control in one trial of a two-choice task, whereby the sensorimotor states that correspond to choice 1 (e.g., pushing button #1) and choice 2 (e.g., pushing button #2) correspond to two alternative actions. In this model, only the higher-level goal (H-goal) is referred to with the label “goal.” In the trade-off between exploration and exploitation, goals tend to fall on the side of exploitation, leaving the impression that explorative activity is not driven by a goal. With regard to the modes of action, we read, “While a strong maintenance of goals helps concentrating on relevant information and suppressing irrelevant information, it increases the probability that this renders a cognitive system too inflexible…” (Hommel, 2015, p. 44). And, “the way a given individual exerts control operations in a given situation can be biased either toward persistence (of goals, preferences, working-memory content, etc.) or toward flexibility” (Hommel and Colzato, 2017, p. 44).

This elegant simplification based on persistence (of a goal) and flexibility, can account for many variations in exploitative activity. With regard to exploration, however, the divorce from goals seems unwarranted. In Figure 1B, we have proposed a revision that regards the hierarchical nature of goals. The sensorimotor states corresponding to choice 1 and 2 are, thus, labeled “L-goal 1” and “L-goal 2.” In this hypothetical trial of the task (Figure 1), choice 1 is the correct action, determined by the current higher-level (H-)goals, which is determined by the task.

Hommel's (2015) model is primarily a model of exploitation, which means it regards exploitation as the default mode of action. Moving from exploitation to exploration is described in terms of two changes: First, the link between the higher-level goal (H-goal) and the lower-level goal (L-goal) is weakened. Second, the inhibitory link between alternative L-goals is weakened. An agent enters the explorative mode once the H-goals do not activate their corresponding L-goals, and once competing L-goals do not inhibit each other. In short, if we regard goals to exist at one level (e.g., H-goal), then it would seem reasonable to describe exploration as goal-free. In line with the dominant understanding of the distinction between exploitative and explorative agency (Hills et al., 2015), and the treatment of goals as a unitary concept, Hommel's model describes exploration as a deviation from exploitation, and thus maintaining the problematic status of explorative behavior as genuine (goal-directed) action.

In the modified version, shown in Figure 1B, we highlight that goals are not an external force that produce imbalance between competing states at the lower levels of the hierarchy of activities (as Figure 1A suggests). Instead, higher-level goals are implemented in terms of specific states at the lower levels. In Figure 1B, an H-goal is characterized in part as the inhibitory relation between L-goal 1 and L-goal 2. The same logic applies to goals at different levels of the hierarchy, highlighted by the inhibitory relation between H-goal 1 and H-goal 2.

**Figure 2:**
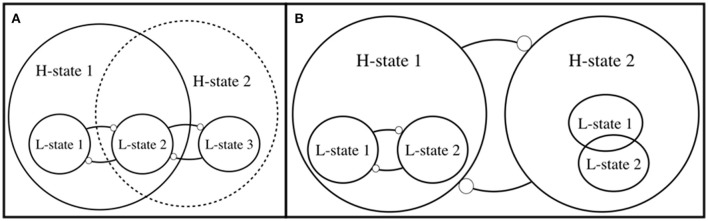
Shows possible relation between relatively higher and relatively lower states in the hierarchy that constitutes an activity. **(A)** Exercise of the same L-state 2 without its close tie to H-state 1 can lead to establishment of the new H-state 2. For instance, the act of speaking in public might be associated with a new H-goal, such as entertaining, persuading, or alerting other people. **(B)** H-state 1 and H-state 2 differ from each other in terms of whether or not two lower-level states, L-state 1 and L-state 2 are in inhibitory relation with each other. For instance, going home from work, once it is not strictly tied to path toward home, can lead to finding opportunities along the way, e.g., a cafe or art gallery. Depending on the level at which we conceive of the states, explorative activity can result in switching from H-state 1 to H-state 2, or it could result in the agent's understanding that she exists in one of the two H-states and not the other. For the sake of simplicity, we have included only two levels in the hierarchy.

Exploration can be characterized in terms of not having the ability or the intention to move toward a *specific* H-goal, although one might move toward a non-specific H-goal. Consider, again, the example of going home from work. When this action becomes more explorative, as you take a new path home, you still rely on a set of skills to fulfill L-level navigation goals. You will also eventually arrive home, but there is now room for other H-goals (a non-specific H-goal, in this example, could be described as “discovering something new, interesting, or fun along the way”). In this sense, we are in agreement with Hommel's (2015) view regarding the loosening of the relation between H- and L-goals, although the consequences of this loosening can be made more explicit (Figure 2). In Figure 2A, L-state 2, which is originally linked only to H-state 1, is shown to be also connectable to H-state 2. In the example shown in Figure 2, H-state 1 consists of an antagonism between L-states 1 and 2 (e.g., driving home and stopping by at art gallery), whereas H-state 2 might consist of a new antagonism between L-states 2 and 3 (e.g., driving home and taking the fastest route). We can conceive of H-state 2, as well as the link between L-state 2 and H-state 2, as a *possibility* that is discovered via exploration.

Another possible consequence of exploration can be expressed in terms of understanding whether certain low-level states are in inhibitory relation. In Figure 2B, we could think of the antagonism between the two H-states in terms of ground for hypothesis testing (e.g., “H-states 1 and 2 cannot both be factual-Which one is?”). In such a state, bringing about a particular L-state, e.g., conducting an experiment and finding out whether L-state 1 and 2 can simultaneously obtain, would be an act of testing whether one lives in a particular world among a set of theoretically *possible worlds*. This applies also to everyday matters, e.g., when one discovers that the possible world in which going home from work and visiting an art gallery co-occur is actualized.

Given the present perspective, individual differences in exploration could be linked to several factors. In a relatively trivial sense, if the pursuit of L-goals is made difficult, then the organism is less likely to rely on them as a means to explore (e.g., a leg injury reduces the likelihood of explorative walking). It is also conceivable that performance difficulty in achieving L-goals might reduce attention to H-goals and, thus, reduce the likelihood of learning associations between L-goals and novel H-goals (Vallacher and Wegner, 1989). There is some evidence against this idea from studies that have shown difficult-to-achieve L-goals do not interfere with the acquisition of new corresponding H-goals (e.g., Gozli et al., 2016). Furthermore, negative emotion associated with facing the unfamiliar could also account for individual differences in explorative action (Rolls, 2000). Exploration tends to be more frequent in children than adults, both in the sense of play in the physical-social environment and in the sense of *pretend play* that extends to the conceptual-hypothetical domain (Piaget, 1954). At the same time, a secure “home base,” e.g., attachment to the primary caregiver, has been shown to account for differences in explorative activity in children (Ainsworth and Bell, 1970). The interplay between emotions, knowledge, and skills in explorative activity merits further study.

## Conclusion

Recognizing the hierarchical organization of goals is crucial in understanding the capacity for exploration. Contrary to the dominant models, we maintain that exploration does not imply absence of goals, but rather goals at the relatively lower levels of the hierarchy, and their saliency in explorative agency. Exploration implies (a) skills for the fulfillment of L-goals, (b) the possibility of new organization between goals at different levels or new ways in which L-goals and H-goals can be linked, and (c) the generative capacity to discover new goals at any level of the hierarchy. In this sense, exploitation is not the default mode of goal-directed activity. Rather, the hierarchy of goals that is expressed in exploitation requires as its necessary condition a history of explorative activity that has shaped the goal hierarchy (Elsner and Hommel, 2001). Just as exploitation corresponds to the possibility of goal-fulfillment, exploration corresponds to the possibility of goal-discovery and the reorganization of goal hierarchies.

## Author contributions

DG conceived of the initial idea and wrote the first draft. DG and ND both wrote and revised the next drafts of the manuscript. DG and ND both approved the final version.

### Conflict of interest statement

The authors declare that the research was conducted in the absence of any commercial or financial relationships that could be construed as a potential conflict of interest.

## Footnotes

^1^The distinction has also been framed in terms of the opposition between narrow *vs*. wide search, and between persistence *vs*. flexibility. The former dichotomy refers to the trade-off between a narrow search, with a more determinate outcome, and a wide search with less determinate (but possibly more rewarding) outcome (Hills et al., 2015). The latter dichotomy refers to the trade-off between persisting on known strategies and being flexible to alternative (potentially more effective) strategies (Hommel, 2015).
